# Super-Resolution Ultrasound Reveals Cerebrovascular Impairment in a Mouse Model of Alzheimer's Disease

**DOI:** 10.1523/JNEUROSCI.1251-23.2024

**Published:** 2024-02-28

**Authors:** Matthew R. Lowerison, Nathiya Vaithiyalingam Chandra Sekaran, Zhijie Dong, Xi Chen, Qi You, Daniel A. Llano, Pengfei Song

**Affiliations:** ^1^Beckman Institute for Advanced Science and Technology, University of Illinois at Urbana-Champaign, Urbana, Illinois 61801; ^2^Departments of Electrical and Computer Engineering, Urbana, Illinois 61801; ^3^Molecular and Integrative Physiology, Urbana, Illinois 61801; ^4^Bioengineering, University of Illinois at Urbana-Champaign, Urbana, Illinois 61801; ^5^Neuroscience Program, University of Illinois at Urbana-Champaign, Urbana, Illinois 61801

**Keywords:** Alzheimer's disease, cerebral blood flow, microbubble, mouse, super-resolution ultrasound

## Abstract

Increasing evidence has suggested a link between cerebrovascular disease and the cognitive impairment associated with Alzheimer's disease. However, detailed descriptions of microvascular changes across brain regions and how they relate to other more traditional pathology have been lacking. Additionally, the efforts to elucidate the interplay between cerebral microvascular function and Alzheimer's disease progression are complicated by the necessity of probing deep-brain structures since early-stage Alzheimer's disease typically involves hippocampal pathology. The purpose of this study was to examine changes in microvascular dynamics in a mouse model of Alzheimer's disease using cohorts that were age-matched to wild-type controls. Data from both sexes were included in this study. Super-resolution ultrasound localization microscopy revealed microvascular functional and structural features throughout the whole brain depth to visualize and quantify. We found that functional decreases in hippocampal and entorhinal flow velocity preceded structural derangements in regional vascular density. Co-registered histological sectioning confirmed the regionalized perfusion deficits seen on ultrasound imaging, which were co-localized with amyloid beta plaque deposition. In addition to providing global vascular quantifications of deep brain structures with a high local resolution, this technology also permitted velocity-profile analysis of individual vessels and, in some cases, allowed for decoupling of arterial and venous flow contributions. These data suggest that microvascular pathology is an early and pervasive feature of Alzheimer's disease and may represent a novel therapeutic target for this disease.

## Significance Statement

Studies of the impact of cerebrovascular pathology on Alzheimer's disease are complicated by the difficulty of imaging deep-brain structures with high fidelity. We demonstrate that ultrasound localization microscopy, a super-resolution acoustic imaging technique, is capable of imaging cerebrovasculature throughout the entire depth of the mouse brain at a microvascular scale. This technology was applied to the 5xFAD mouse model of Alzheimer's disease, where it was found that 5xFAD animals have significant impairments in vascular function in the entorhinal cortex and hippocampus compared to age matched controls at the 3-month timepoint. Structural derangements in cerebrovasculature were only observed at the 6-month-old timepoint, with a maintained impairment in vascular function. These findings suggest that microvascular pathology occurs early in the Alzheimer's disease cascade.

## Introduction

Alzheimer's disease (AD) is the leading cause of dementia in older adults. Approximately 6 million Americans are impacted by this disease, and this number is projected to more than double to 14 million by 2060 (Matthews et al., 2019), leading to the loss of quality of life in a substantial and growing population. Unfortunately, there are currently no highly effective preventative or therapeutic strategies to combat this disease, signaling an urgent need to improve early diagnosis and therapeutic options to help prevent, treat, and alleviate AD (HHS, 2021).

The current hallmarks of AD are amyloid beta (Aβ) deposition and abnormal accumulations of neurofibrillary tau protein (Bloom, 2014), motivating substantial research effort centered on the amyloid hypothesis (Glenner and Wong, 1984; Hardy and Allsop, 1991; Karran et al., 2011) of AD. However, additional pathophysiological processes likely also contribute to AD. Increasing evidence has suggested a link between cerebrovascular disease (CVD) and cognitive impairment and other symptoms of patients with AD (de la Torre, 1994), with vascular dysfunction identified as an early and nearly ubiquitous feature of AD (Iadecola, 2004; Kisler et al., 2017). However, CVD and AD share several risk factors such as hypertension (Bellew et al., 2004), diabetes (Ahtiluoto et al., 2010), and obesity (Kivipelto et al., 2005), making it unclear whether cerebrovascular deficiency and AD pathology have additive effects on cognitive decline or if CVD accelerates existing AD-associated pathology. The vascular hypothesis of AD (de la Torre and Mussivand, 1993), details a vicious cycle between vascular dysfunction and AD pathology: cerebrovascular dysregulation initiates a cascade of blood flow reduction and neuronal dysfunction leading to the accumulation of Aβ, which in turn aggravates further vascular dysregulation by triggering hypoperfusion, vasoconstriction, and breakdown of the blood-brain barrier (Kisler et al., 2017). These cerebrovascular impairments worsen Aβ clearance, thereby further accelerating AD pathology. Understanding and overcoming this cycle is essential for developing effective AD therapies and interventions.

Despite CVD's profound impact on AD development and progression, the exact mechanisms and relationships underlying a vascular hypothesis of AD remains difficult to establish due to the requirements of high-fidelity and dynamic characterization of microvascular impairments. Furthermore, early-stage AD typically involves hippocampal atrophy (Schuff et al., 2009), which complicates most efforts to elucidate the interplay between cerebral microvascular function and AD progression as it necessitates the probing of deep-brain structures at a resolution sufficient to image microvasculature – an intractable set of requirements for most medical imaging modalities. Histological analysis of AD-affected brain sections can give insight into the structural correspondence between microvascular density and Aβ deposition but lacks critical information on impairments in blood flow dynamics that likely precede vascular remodeling. Ultrasound localization microscopy (ULM) is an emerging super-resolution ultrasound technique (Christensen-Jeffries et al., 2015; Errico et al., 2015) with a theoretical resolution limit at the capillary-scale without sacrificing imaging penetration depth (Desailly et al., 2015), which may permit *in vivo* characterization of microvessels that have been reported to contain pathology in the brains of AD patients and in AD rodent models (Nortley et al., 2019). ULM is readily translatable to clinical systems (Demené et al., 2021), and has been repeatedly demonstrated to provide microvessel reconstructions of the entire depth of the brain (Couture et al., 2018), including several studies focused on mouse brain imaging (Nouhoum et al., 2021; Demeulenaere et al., 2022).

Here, we apply ULM to a 5xFAD mouse model of AD with age-matched controls from two different stages of AD progression (3- and 6-month-old cohorts). We chose these ages because microvascular changes are seen early during the AD pathological cascade (Iturria-Medina et al., 2016; Apátiga-Pérez et al., 2022) and because these ages represent earliest stages of pathology in these mice. We examine global hippocampal, entorhinal, and isocortex alterations in the microvascular density and blood flow velocity from these two cohorts of animals. The resulting super-resolved whole-brain cross-sectional images are then compared to co-registered histological sections of vascular staining and Aβ deposition. We find that microvascular functional changes precede microvascular structural changes and are most prominent in the hippocampus and entorhinal cortex. These data point to an early and pervasive role for microvascular pathology in AD and introduce a new tool – ULM – to investigate microvascular pathology in AD mouse models.

## Materials and Methods

### Animal model and ethics

All procedures conducted on mice presented in this manuscript were approved by the Institutional Animal Care and Use Committee (IACUC) at the University of Illinois Urbana-Champaign (protocol # 22033). All experiments were performed in accordance with these IACUC guidelines. Mice were housed in an animal care facility approved by the Association for Assessment and Accreditation of Laboratory Animal Care. Every attempt was made to minimize the number of animals used and to reduce suffering at all stages of the study. Reporting in this manuscript follows the recommendations of the ARRIVE guidelines (Percie du Sert et al., 2020).

5xFAD transgenic mice overexpress mutant human Aβ (A4) precursor protein 695 (APP) with the Swedish (K670N, M671L), Florida (I716V), and London (V717I) Familial AD (FAD) mutations along with human presenilin 1 (PS1) harboring two FAD mutations, M146L and L286V. 5xFAD mice (MMRRC Strain # 034840-JAX, Jackson laboratory) were crossbred and generated with B6FJLF1/J (Stock No: #100012, Jackson laboratory) mice. Genotyping was confirmed using Transnetyx service. The sequence information for the 5xFAD is probe huPSEN1 Tg Forward Primer: GGCTACCATTAAGTCAGTCAGCTTT, Reverse Primer: CCCACAGTCTCGGTATCTTCTG, Reporter 1: ATGGGCAGCTAATCTA, for APPsw Tg, Forward Primer: CCGACATGACTCAGGATATGAAGTT, Reverse Primer: CCTTTGTTTGAACCCACATCTTCTG, Reporter 1: AAAGAACACCAATTTTTG and for the probe Chr3-6 WT, Forward Primer: GGAGGTGGACTTCACAAATTCC, Reverse Primer: GGGTGACCCTCTAGCAAGTC, Reporter 1: CTGTTGGGCATCTCATCC.

Both 5xFAD and control littermates of either sex, aged 3- and 6-months old, were used in this study. Littermate control animals of the same age were used. Animal cohort sizes were 17 in the 3-month group (5xFAD = 8, WT = 9; *N*_Female_ = 13), and 24 in the 6-month group (5xFAD = 13, WT = 11; *N*_Female_ = 12), with datasets excluded if the animal died prematurely during imaging. Animal weights and heart rates are summarized in Table 1.

**Table 1.** Animal physiological information

		Count (*N*)	Weight (g)	Heart rate (BPM)
3-month		17	26.8 ± 3.5	416.3 ± 28.2
WT		9	27.7 ± 4.0	414.1 ± 32.2
	Male	3	32.8 ± 1.7	381.1 ± 7.0
	Female	6	25.1 ± 1.6	430.6 ± 26.5
5xFAD		8	25.8 ± 2.6	418.7 ± 23.2
	Male	1	29.0 ± NA	421.6 ± NA
	Female	7	25.4 ± 2.5	418.3 ± 24.8
6-month		24	27.9 ± 6.7	424.1 ± 37.0
WT		11	28.2 ± 6.3	421.4 ± 33.4
	Male	6	29.4 ± 3.4	398.3 ± 26.1
	Female	5	26.7 ± 8.6	449.1 ± 15.1
5xFAD		13	27.8 ± 7.0	426.3 ± 39.9
	Male	6	33.5 ± 6.2	418.0 ± 48.0
	Female	7	22.9 ± 2.6	433.4 ± 31.0

### Craniotomy preparation for ultrasound imaging

**Figure 1.**
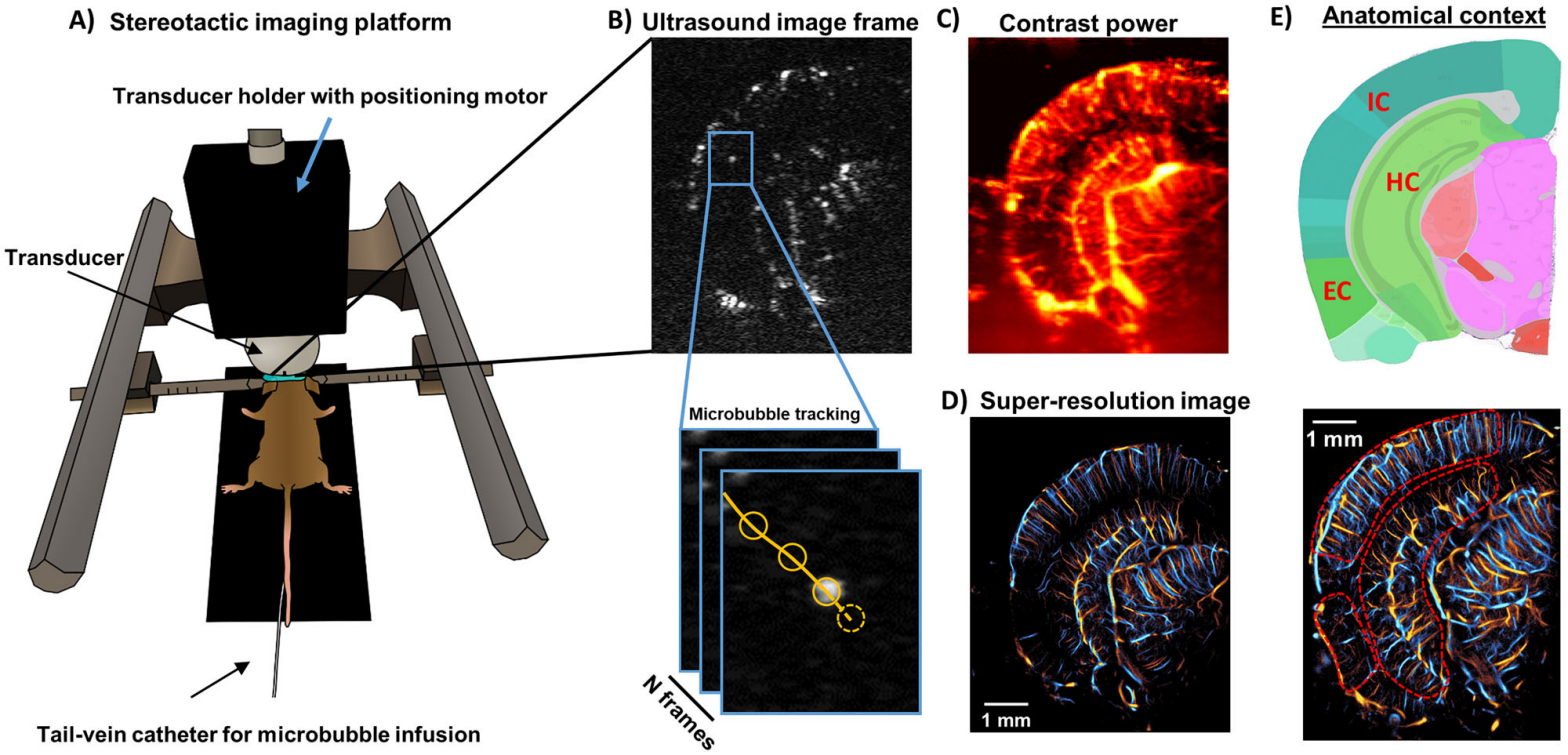
Overview of ULM imaging acquisition. ***A***, Post-craniotomy animals were secured to a stereotaxic imaging frame via ear-bars, and an ultrasound transducer with high-precision motor was positioned to acquire the anatomical imaging plane of interest. ***B***, An example imaging frame of microbubble contrast-enhanced data taken from a coronal cross-section of the brain. Individual microbubble centroids were localized and tracked to produce blood flow trajectories (orange line). ***C***, A conventional contrast-enhanced power Doppler image of the mouse brain, with (***D***). A super-resolved directional flow ULM image of the same dataset as in (***C***), demonstrating finer detail of the cerebral microvasculature. ***E***, A corresponding Allen Brain Atlas image used to identify brain regions.

Mouse anesthesia was induced using a gas induction chamber supplied with 4% isoflurane mixed with medical oxygen. Mice were then placed in a stereotaxic frame with nose cone supplying 2% isoflurane with oxygen for maintenance, with 1% lidocaine intradermally injected into the scalp to supplement anesthesia. The mouse's head was secured to the stereotaxic imaging stage via ear bars (Fig. 1*A*). The scalp of the mouse was removed with surgical scissors, and a cranial window was opened on the left side of the skull using a rotary Dremel tool. The cranial window started at the sagittal suture and extended laterally to expose the lateral expanse of the cerebral cortex.

### Contrast-enhanced ultrasound imaging of mouse brain

A Verasonics Vantage 256 research ultrasound system (Verasonics Inc., Kirkland, WA) was used for all ultrasound imaging in this study. A L35-16vX transducer (Verasonics) was secured to the stereotaxic imaging frame via a 3D-printed transducer holder (Fig. 1*A*) with attached high-precision translation motor (VT-80 linear stage, Physik Instrumente, Auburn, MA). The transducer was oriented to produce a coronal anatomical section of the brain (Fig. 1*B*). Acoustic contact gel was applied directly to the surface of the mouse brain. Once acoustic coupling was established, the motorized stage was adjusted by 0.1 mm increments to find an imaging plane which contained a cross-section view of hippocampus and entorhinal cortex (Fig. 1*C*). This was 3.0 mm caudal to the bregma. The imaging field of view was then adjusted laterally/axially to cover the entire half of the mouse brain in this anatomical position. Two additional imaging planes, one 0.5 mm caudal to this center position and one 0.5 mm rostral to it (bregma −3.5 mm and bregma −2.5 mm, respectively), were also selected for imaging to provide additional sampling of hippocampal and entorhinal cortex vasculature.

The tail vein of each mouse was cannulated with a 30-gauge catheter and vessel patency was confirmed with a 0.1 ml injection of sterile saline. Freshly activated DEFINITY® was infused via the tail vein catheter at a rate of 10 µl/min using a programmable syringe pump (NE-300, New Era Pump Systems Inc., Farmingdale, NY). The microbubble solution was mixed every 5 min using a magnetic stirrer to maintain a constant microbubble concentration during the experiment. Ultrasound imaging was performed at a center frequency of 20 MHz, using 9-angle plane wave compounding (1-degree increments) with a post-compounding frame rate of 1,000 Hz (Fig. 1*B*). A total of 64 s of data (64,000 frames) were acquired for each imaging plane. Ultrasound data were saved as beam-formed in-phase quadrature (IQ) datasets for off-line processing in MATLAB (The MathWorks, Natick, MA; version R2019a).

### Reconstruction of super-resolution brain vascular images

Singular value decomposition (SVD) clutter filtering was applied to extract microbubble signals from tissue background from each IQ dataset (Desailly et al., 2017; Song et al., 2018b; Huang et al., 2020; Lowerison et al., 2020b), with the singular value threshold determined adaptively (Song et al., 2017a). An adaptive SVD threshold was selected over conventional fixed threshold processing to better account for variable tissue motion in the experimental dataset, which can shift the optimal tissue/microbubble subspace delineation (Baranger et al., 2018). A noise-equalization profile (Song et al., 2017b) was applied to equalize the microbubble signal intensity through the entire depth of imaging. A 2D Gaussian function was empirically fit to the axial and lateral dimensions of manually selected microbubbles to represent the point-spread function (PSF) of the system. Microbubble separation (Huang et al., 2020) was applied to post-SVD-filtered IQ data, and then each IQ data subset was spatially interpolated to an isotropic 4.928 µm axial/lateral pixel dimension using 2D spline interpolation (Song et al., 2018a). Normalized 2D cross-correlation was performed on this interpolated dataset with the empirical PSF to identify candidate microbubble localizations (Fig. 1*B*). Pixels with a low cross-correlation coefficient were excluded via a threshold (Huang et al., 2020; Lowerison et al., 2020b; Tang et al., 2020), and microbubble centroids were localized with the MATLAB built-in “imregionalmax.m” function. Frame-to-frame microbubble centroid pairing and trajectory estimation was performed using the uTrack algorithm (Jaqaman et al., 2008). A minimum microbubble trajectory length of 10 frames (i.e., 10 ms) was applied to the super-resolution reconstructions presented in this study. Each acquisition was then accumulated to produce a final ULM vascular reconstruction of the brain vasculature (Fig. 1*D*), plotted at an isotropic 4.928 µm axial/lateral pixel dimension.

### Ultrasound image analysis

**Figure 2.**
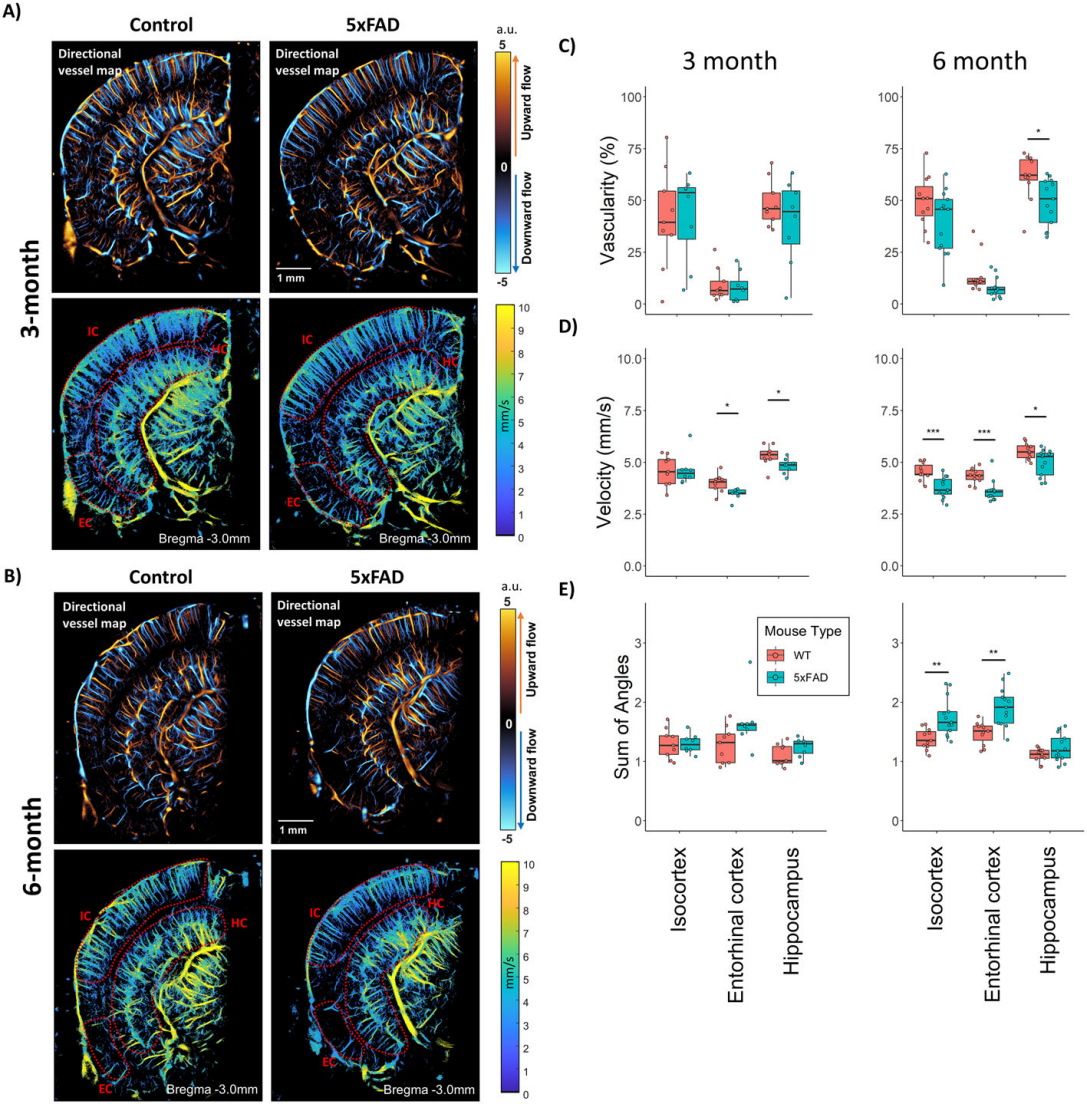
Regional anatomy analysis with ULM. Example directional flow and velocity maps taken from a WT control (left) and 5xFAD (right) mouse in this study, for (***A***) 3-month and (***B***) 6-month animals. Segmentations were placed around dorsal isocortex (IC), entorhinal cortex (EC), and hippocampus (HC) ROIs for analysis, as demonstrated on the velocity maps. ***C***, The 6-month-old 5xFAD cohort demonstrated significant decreases in hippocampus vascularity in comparison to WT controls. No decrease in vascularity was noted for the 3-month cohort. ***D***, A significant decrease in overall median velocity was noted in the entorhinal cortex and the hippocampus for the 3-month 5xFAD cohort. This decrease in velocity was sustained in the 6-month-old 5xFAD cohort, with significance found in all three ROIs. ***E***, The 5xFAD cohorts had increased tortuosity, as measured by the SOAM, in the isocortex and entorhinal cortex in the 6-month cohort, with evidence of early increased tortuosity in the entorhinal cortex of the 3-month animals (Multiplicity adjusted *p* values: *p < 0.05, **p < 0.01, ***p < 0.001).

Anatomical regions (Fig. 2) were manually segmented by placing Bezier control vertices on the border of the region of interest (ROI), informed by the Allen Brain Atlas (https://mouse.brain-map.org/static/atlas), and by interpolating between each vertex using Hobby's algorithm (Hobby, 1986). Registration between ULM vascular images and the Allen Brain Atlas was performed manually. Three anatomical regions of interest were selected: the isocortex (encompassing portions of the auditory and visual cortices), the entorhinal cortex, and the hippocampal region. Brain vascularity within each ROI was calculated by binarization of super-resolution vessel maps to determine the percentage of cross-sectional area that contained microbubbles. Blood vessel velocity was determined for every microbubble track directly from the frame-to-frame displacement of detected microbubble centroids. Vascular tortuosity, as measured by the sum of angles metric (SOAM), was calculated using the algorithm described by Shelton et al. (2015) for every microbubble trajectory and averaged temporally across all data accumulations. Blood velocity profiles were determined by manually selecting vessel cross-sections on the final ULM accumulation, and then extracting all raw velocity trajectories that crossed the line segmentation (Fig. 5). Parabolic profiles were fit to the raw maximum velocity estimates using MATLAB.

### FITC dextran perfusion and tissue processing

Following ULM imaging, FITC-dextran (70,000 kDa; 0.1 ml 50 mg/ml; Sigma, St. Louis, MO, USA) was administered intravenously through the tail vein catheter. Animals were sacrificed 5 min after the complete injection of FITC-dextran to allow recirculation of the indicator. Brains were rapidly removed and placed in 4% paraformaldehyde (PFA) in phosphate-buffered saline (PBS) at 4°C for 24–48 h and then cryoprotected in ascending series of 10%, 20%, 30% sucrose in PBS for another 24–36 h at 4°C. Each brain was embedded and cut into 40-μm-thick sections on a cryostat.

### Immunostaining

Sections were washed in Tris-buffered saline (TBS) 3 times for 10 min each. Sections were then incubated for 30 min in a solution of 0.3% Triton X-100 in TBS to enhance membrane permeability. The sections were then transferred to a blocking solution consisting of 0.3% Triton X-100% and 5% normal goat serum and 3% bovine serum albumin in TBS and incubated for 60 min. The primary antibody solution consisted of 1:1,000 purified anti-β-amyloid, 1–16 antibody (catalog# SIG-39320, Biolegend) in 0.3% Triton X-100% and 5% normal goat serum and 3% bovine serum albumin in TBS. Sections were incubated in this solution overnight and rinsed in three changes of the Triton X-100 in TBS the following day. The sections were then transferred to a secondary antibody solution and incubated at room temperature for 2 h. This solution consisted of 1:100 Alexa Fluor 568-conjugated goat anti-mouse secondary antibody (catalog #A-11004, Invitrogen). Following a final series of washes in TBS, the sections were mounted on gelatin-coated slides and coverslipped with an anti-fade mounting medium (Vectashield; Vector Laboratories).

### Histology imaging and analysis

Sections used for histochemical analysis were imaged using a Confocal - Zeiss LSM 710 - Multiphoton Microscope and Zen blue software. For each tissue section, 40× mosaic Z-stacks were taken throughout the entire depth and *x*–*y* plane of the brain. Green-colored FITC images were obtained with a 488 nm laser and emission at 491–561 nm. Red-colored Aβ images were obtained with a 561 nm laser with emission at 565–640 nm. The stacks were collapsed into 2D maximum intensity projections and tiled into a single image using Zen blue software.

For quantification of amyloid and vascular densities, wide-field images were obtained using an Olympus IX71 microscope using a 5× and 10× objective. Images for the quantification for vasculature were taken with a U-MF2-GFP cube with excitation: 472 ± 30 nm and emission: 520 ± 35 nm. For amyloid deposition imaging, a U-MF/XL red cube with excitation: ET 545 ± 30 nm and emission: 593 ± 30 nm was used.

ROI segmentations of the anatomy of interest (isocortex, entorhinal cortex, or hippocampus) were manually placed using ImageJ. The ROI coordinates were then imported into MATLAB for quantitative analysis of Aβ positive staining to measure amyloid burden, and FITC staining as a measure of vascular density. An adaptive threshold, based on the median signal value within a sliding search window, was applied separately to both the Aβ channel and the FITC channel to segment out the respective positive signal. The use of an adaptive threshold was necessary to correct for uneven illumination and vignetting artifacts. An additional morphometric exclusion mask was applied to the FITC channel due to the presence of signal from Aβ fluorescence.

### Experimental design and statistical analyses

All statistical analysis was performed in the R programming language (R Core Team, 2019), and all graphs were generated using the ggplot2 package (Wickham, 2016). All repeated measurements from the same animal (e.g., multiple histological sections, multiple imaging planes, multiple vessel line profiles) were averaged into a single replicate before analysis. An analysis of variance (ANOVA) was applied to test for statistical significance between WT and 5xFAD mouse brain anatomy, with a correction for multiplicity performed using the Benjamini and Hochberg step-up procedure to control for false-discovery rate (Benjamini and Hochberg, 1995). A corrected *p* < 0.05 was considered as statistically significant, and all *p* values in figures used these corrected values. Both the uncorrected and corrected *p* values are reported in the main text. Two-way ANOVAs were performed to analyze the effect of genotype and rostro-caudal location on ULM vascularity metrics, as well as between animal genotype and sex. A two sample Kolmogorov–Smirnov test was used to determine that sex-based ULM metrics were statistically not significantly different. All summary statistics in text are presented as the mean ± standard deviation.

## Results

### Super-resolution ULM provides microvascular fidelity over whole brain depth

A diagrammatic example of the imaging platform and protocol used in this experiment is demonstrated in Figure 1*A*. After craniotomy, the anesthetized mouse was secured via ear-bars to a stereotactic imaging frame with connected ultrasound transducer and high precision motor for imaging cross-section placement. After reaching a steady-state concentration, the microbubble contrast agent in the mouse brain was visible as distinct, flowing, Gaussian-shaped features in the imaging acquisition following SVD-filtering (Fig. 1*B*). These diffraction-limited microbubble features could be extracted and localized at a sub-diffraction precision and tracked frame-to-frame to produce flow trajectories yielding both the location and velocity of the cerebro-microvasculature. Conventional contrast power processing (Fig. 1*C*) demonstrates the blood volume of the mouse brain but lacks the ability to distinguish microvasculature. After accumulating all the microbubble trajectories, the final ULM image (Fig. 1*D*) demonstrates well defined microvascular reconstructions for both superficial (cortical) and deep-brain structures (e.g., hippocampus) and includes flow direction and velocity information, with a corresponding Allen Brain Atlas (Fig. 1*E*) providing anatomical context for ULM segmentation and quantification.

### The 5xFAD cohort demonstrates global hypo-perfusion in deep brain structures in comparison to WT control

Super-resolution ULM directional vessel maps and velocity maps were reconstructed for the WT and 5xFAD cohorts in this study, as demonstrated in Figure 2*A,B*. Manual segmentations were performed on these imaging cross-sections with the aid of the Allen Brain Atlas to produce ROIs corresponding to the dorsal isocortex (region IC), the entorhinal cortex (region EC), and the hippocampus (region HC). Poor vascular reconstruction was noted for the most lateral portion of the cortex, due to interference from strong acoustic scattering from the ear bar or acoustic shadowing from the remaining portion of the lateral skull. Quantification of regional anatomy measures of vascularity, median flow velocity, and the sum-of-angles metric (a measure of tortuosity) are demonstrated in Figure 2*C–E*, where the results are the average of the three imaging planes.

The vascularity index is a measure of the total percentage of the ROI, a metric that is analogous to microvascular density (Fig. 2*C*). No statistically significant difference in ULM vascularity index was found between the WT and 5xFAD animals in the 3-month cohort for the anatomical regions examined: Isocortex (WT = 41.4 ± 24.2% vs 5xFAD = 42.6 ± 21.6%, ANOVA *F*_(1,15)_ = [0.012], *p* = 0.91, *p*_corrected_ = 0.91), entorhinal cortex (WT = 9.4 ± 7.8% vs 5xFAD = 8.2 ± 7.3%, ANOVA *F*_(1,15)_ = [0.097], *p* = 0.76, *p*_corrected_ = 0.91) and hippocampus (WT = 48.5 ± 11.1% vs 5xFAD = 39.8 ± 20.4%, ANOVA *F*_(1,15)_ = [1.218], *p* = 0.28, *p*_corrected_ = 0.86). For the 6-month cohort, the isocortex demonstrated a non-significant trend toward a decrease in the 5xFAD animals in comparison to WT (WT = 49.6 ± 12.4% vs 5xFAD = 39.1 ± 15.7%, ANOVA *F*_(1,22)_ = [3.231], *p* = 0.086, *p*_corrected_ = 0.086). Likewise, the entorhinal cortex demonstrated a trend toward a decrease in the 5xFAD animals, but this was not found to be significant after correcting for multiple comparisons (WT = 14.1 ± 9.1% vs 5xFAD = 7.9 ± 4.9%, ANOVA *F*_(1,22)_ = [4.471], *p* = 0.046, *p*_corrected_ = 0.069). The most profound decrease in ULM vascularization index was found for the hippocampus of the 6-month cohort (WT = 61.2 ± 10.9% vs 5xFAD = 49.2 ± 11.3%, ANOVA *F*_(1,22)_ = [7.005], *p* = 0.015, *p*_corrected_ = 0.045).

The 3-month cohort had significant decreases in flow velocity (Fig. 2*D*) for the entorhinal cortex (WT = 3.99 ± 0.47 mm/s vs 5xFAD = 3.48 ± 0.26 mm/s, ANOVA *F*_(1,15)_ = [8.385], *p* = 0.011, *p*_corrected_ = 0.033) and the hippocampus (WT = 5.32 ± 0.50 mm/s vs 5xFAD = 4.82 ± 0.36 mm/s, ANOVA *F*_(1,15)_ = [5.472], *p* = 0.034, *p*_corrected_ = 0.05). No significant difference in ULM flow velocity for the isocortex was found between the WT and 5xFAD animals in the 3-month cohort (WT = 4.60 ± 0.71 mm/s vs 5xFAD = 4.62 ± 0.72 mm/s, ANOVA *F*_(1,15)_ = [0.002], *p* = 0.965, *p*_corrected_ = 0.965). We found that these decreases in velocity were sustained in the 6-month group, with all anatomical ROIs demonstrating a significant reduction in flow speed. The largest decrease was seen in the isocortex (WT = 4.55 ± 0.41 mm/s vs 5xFAD = 3.77 ± 0.51 mm/s, ANOVA *F*_(1,22)_ = [16.847], *p* < 0.001, *p*_corrected_ < 0.001) followed by the entorhinal cortex (WT = 4.35 ± 0.35 mm/s vs 5xFAD = 3.61 ± 0.51 mm/s, ANOVA *F*_(1,22)_ = [16.132], *p* < 0.001, *p*_corrected_ < 0.001) and finally the hippocampus (WT = 5.54 ± 0.39 mm/s vs 5xFAD = 4.97 ± 0.64 mm/s, ANOVA *F*_(1,22)_ = [6.522], *p* = 0.018, *p*_corrected_ = 0.018).

The sum-of-angles tortuosity metric (SOAM) (Fig. 2*E*) was found to have a nonsignificant trend toward being higher in the 3-month 5xFAD animals in the entorhinal cortex (WT = 1.28 ± 0.31 a.u. vs 5xFAD = 1.66 ± 0.45 a.u., ANOVA *F*_(1,15)_ = [4.315], *p* = 0.055, *p*_corrected_ = 0.165), and was not found to be elevated in either the isocortex (WT = 1.3 ± 0.25 a.u. vs 5xFAD = 1.29 ± 0.16 a.u., ANOVA *F*_(1,15)_ = [<0.0001], *p* = 0.98, *p*_corrected_ = 0.98) or the hippocampus (WT = 1.11 ± 0.18 a.u. vs 5xFAD = 1.24 ± 0.16 a.u., ANOVA *F*_(1,15)_ = [2.57], *p* = 0.13, *p*_corrected_ = 0.195). For the 6-month-old 5xFAD animals SOAM was found to be elevated in the isocortex (WT = 1.38 ± 0.17 a.u. vs 5xFAD = 1.75 ± 0.32 a.u., ANOVA *F*_(1,22)_ = [11.39], *p* = 0.003, *p*_corrected_ = 0.005) and the entorhinal cortex (WT = 1.48 ± 0.19 a.u. vs 5xFAD = 1.92 ± 0.32 a.u., ANOVA *F*_(1,22)_ = [15.97], *p* = <0.001, *p*_corrected_ = 0.018). No significant difference was found for the hippocampus (WT = 1.1 ± 0.12 a.u. vs 5xFAD = 1.22 ± 0.23 a.u., ANOVA *F*_(1,22)_ = [2.459], *p* = 0.131, *p*_corrected_ = 0.131).

In summary, the early stage 5xFAD animals show little-to-no structural vascular impairments in any of the examined regions, with the older 5xFAD cohort demonstrating a subtle trend toward reduced vascularization in AD hotspots (hippocampus and entorhinal cortex). In contrast, the 6-month-old 5xFAD cohort presented evidence for impairment in vascular functionality, particularly as measured by flow velocity, in all regions. The 3-month cohort demonstrated a less pronounced impairment of functional metrics in the hippocampus and entorhinal cortex. Together, these results imply that there may be a functional impairment of vasculature (velocity, tortuosity) in the 5xFAD model which precedes structure changes (vascularity) of the cerebrovascular network.

### ULM metrics were not correlated to mouse physiological indices

Global measures of cerebral blood flow may be susceptible to several confounding factors, such as animal sex, weight, and heart rate, which could potentially bias the differences between 5xFAD mice and WT controls noted in Figure 2. To mitigate this concern, we investigated the correlation between animal physiology and ULM imaging metrics. Average animal weight was not found to be different between the WT and 5xFAD cohorts for either age group (3-month: WT = 26.8 ± 3.5 g vs 5xFAD = 27.9 ± 6.7 g, ANOVA *F*_(1,15)_ = [1.046], *p* = 0.323, *p*_corrected_ = 0.646), with an expected upward trend in weight for the older group (6-month: WT = 27.8 ± 7.0 g vs 5xFAD = 28.2 ± 6.7 g, ANOVA *F*_(1,22)_ = [0.021], *p* = 0.885, *p*_corrected_ = 0.885). As expected, male animals were found to be significantly heavier than female animals, with a two-way ANOVA finding a significant effect for animal sex (*F*_(1,37)_ = 19.96, *p* < 0.001) but not for animal genotype (*F*_(1,37)_ = [0.007], *p* = 0.934), or for the interaction effect (*F*_(1,37)_ = [1.785], *p* = 0.190). We found no significant correlations between any ULM metric and animal weight in this study (WT: vascularity (*R* = −0.069, *p* = 0.6), velocity (*R* = −0.12, *p* = 0.35), SOAM (*R* = 0.094, *p* = 0.47); 5xFAD: vascularity (*R* = 0.066, *p* = 0.61), velocity (*R* = 0.037, *p* = 0.77), SOAM (*R* = −0.054, *p* = 0.67)).

Average animal heart rate, measured via the pulsatility in power Doppler signal, was also not found to be different between the WT and 5xFAD cohorts for either age group (3-month: WT = 414.1 ± 32.2 BPM vs 5xFAD = 418.7 ± 23.2 BPM, ANOVA *F*_(1,15)_ = [0.104], *p* = 0.751, *p*_corrected_ = 0.758; 6-month: WT = 421.4 ± 33.4 BPM vs 5xFAD = 426.3 ± 39.9 BPM, ANOVA *F*_(1,22)_ = [0.097], *p* = 0.758, *p*_corrected_ = 0.758). Female animals were found to have a higher heart rate compared to male animals (sex effect *F*_(1,37)_ = [7.920], *p* = 0.007; genotype *F*_(1,37)_ = [0.047], *p* = 0.829; interaction *F*_(1,37)_ = 3.882, *p* = 0.056), which has been previously reported in literature (Fischer et al., 2021). As with animal weight, we found no significant correlations between ULM metrics and animal heart rate (WT: vascularity (*R* = 0.034, *p* = 0.8), velocity (*R* = 0.025, *p* = 0.85), SOAM (*R* = 0.063, *p* = 0.63); 5xFAD: vascularity (*R* = 0.15, *p* = 0.24), velocity (*R* = 0.13, *p* = 0.31), SOAM (*R* = −0.2, *p* = 0.12)).

There is a growing body of literature reporting sex-related differences in pathology for the 5xFAD mouse model (Forner et al., 2021; Poon et al., 2023). As such, we investigated the differences between genotype-matched cross-sex ULM metrics for both WT and 5xFAD animals using a Kolmogorov–Smirnov test to determine if the two sets of samples (male and female) are not significantly different. For ULM vascularity, we found that 3-month WT animals had a *D*_(8)_ = 0.33, *p* = 0.98, and the 3-month 5xFAD animals had a *D*_(7)_ = 0.71, *p* = 0.75. For 6-month cohorts, the cross-sex KS test yielded *D*_(10)_ = 0.3, *p* = 0.89 for WT animals, and *D*_(12)_ = 0.52, *p* = 0.27 for 5xFAD animals. For ULM velocity, the 3-month WT animals had *D*_(8)_ = 0.5, *p* = 0.68, and the 5xFAD animals had a *D*_(7)_ = 0.86, *p* = 0.5, with the 6-month cohort having *D*_(10)_ = 0.4, *p* = 0.68 for WT animals, and *D*_(12)_ = 0.36, *p* = 0.71 for 5xFAD animals. Finally the SOAM metric had *D*_(8)_ = 0.16, *p* = 0.99 for 3-month WT, and *D*_(7)_ = 0.71, *p* = 0.75 for 3-month 5xFAD. The 6-month cohort was *D*_(10)_ = 0.43, *p* = 0.59 for WT animals, and *D*_(12)_ = 0.4, *p* = 0.53 for 5xFAD animals. Given that the genotype-matched cross-sex ULM metrics were found to be not statistically significantly different even for the 6-month cohort, we opted to group both sexes together for subsequent analyses of ULM metrics.

### ULM metrics of flow impairment in the hippocampus are more pronounced in caudal imaging planes for the older cohort

**Figure 3.**
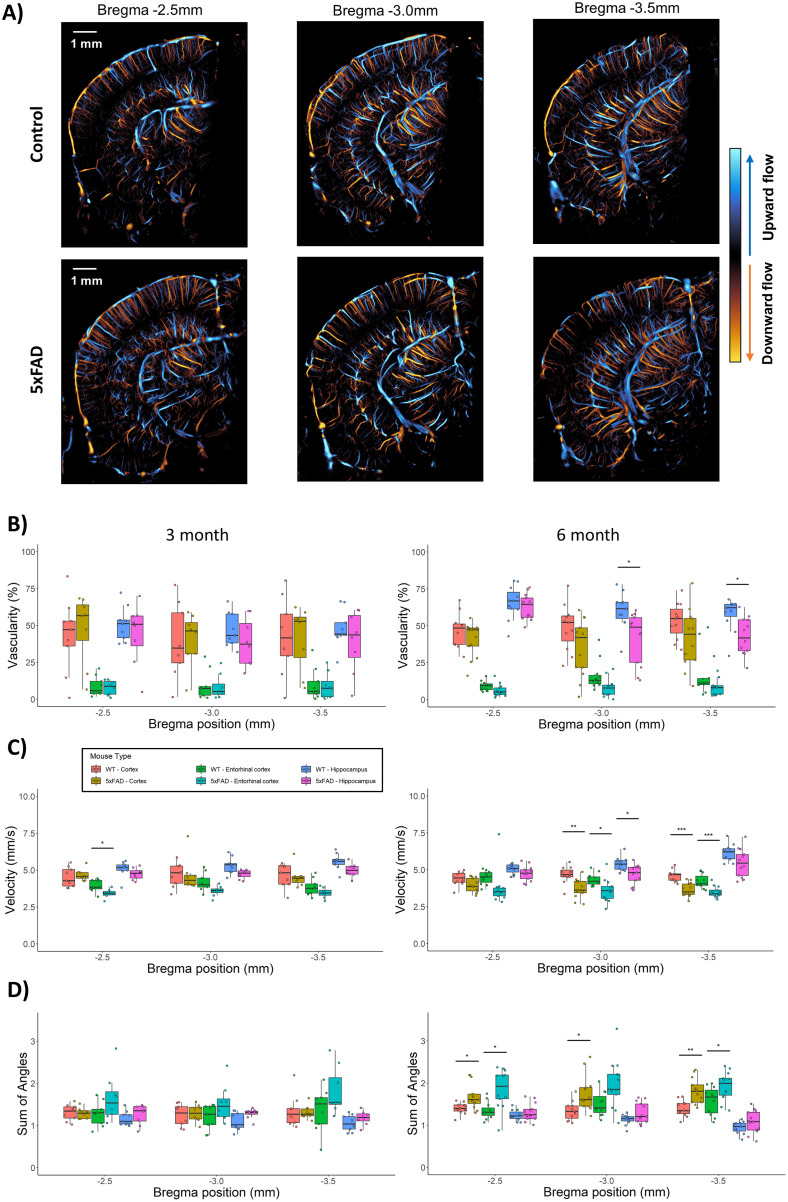
Analysis of ULM metrics and anatomical position. ***A***, Example ULM imaging planes from representative WT/5xFAD animals, with corresponding positions relative to the bregma. Boxplots of ULM metrics from all ROIs and each bregma position for both the 3 and 6 month WT/5xFAD cohorts for (***B***) vascularity (***C***) velocity and (***D***) the sum of angles metric.

In lieu of aggregating the three anatomical imaging planes (Fig. 2*A*), it is also possible to quantify each relative-to-bregma position separately to investigate the posterior to anterior distribution of ULM quantification metrics, as demonstrated in Figure 3. We found that the most caudal imaging section suggested more impairment in ULM metrics in the hippocampus of the 6-month cohort. This observation is consistent with the finding that the caudal hippocampus has a larger burden of plaque (Forner et al., 2021). Two-way ANOVAs were performed to analyze the effect of genotype and rostro-caudal location on ULM vascularity metrics for all anatomical regions.

For the 3-month cohort, we found no statistically significant effect between ULM vascularity and imaging plane or genotype for the isocortex (Position: *F*_(2,45)_ = [0.275], *p* = 0.761; Genotype: *F*_(1,45)_ = [0.104], *p* = 0.749; Interaction: *F*_(2,45)_ = [0.054], *p* = 0.947), entorhinal cortex (Position: *F*_(2,45)_ = [0.089], *p* = 0.915; Genotype: *F*_(1,45)_ = [0.155], *p* = 0.695; Interaction: *F*_(2,45)_ = [0.002], *p* = 0.993), or hippocampus (Position: *F*_(2,45)_ = [0.436], *p* = 0.649; Genotype: *F*_(1,45)_ = [2.970], *p* = 0.092; Interaction: *F*_(2,45)_ = [0.020], *p* = 0.9807). For ULM velocity, there were no significant effects found for the isocortex (Position: *F*_(2,45)_ = [0.063], *p* = 0.939; Genotype: *F*_(1,45)_ = [0.005], *p* = 0.943; Interaction: *F*_(2,45)_ = [0.135], *p* = 0.874). Both the entorhinal cortex and hippocampus demonstrated a significant effect for the animal genotype, but not the imaging plane. Entorhinal cortex (Position: *F*_(2,45)_ = [1.360], *p* = 0.26; Genotype: *F*_(1,45)_ = [16.79], *p* < 0.001; Interaction: *F*_(2,45)_ = [0.242], *p* = 0.786), hippocampus (Position: *F*_(2,45)_ = [2.726], *p* = 0.076; Genotype: *F*_(1,45)_ = [14.07], *p* < 0.001; Interaction: *F*_(2,45)_ = [0.381], *p* = 0.685). The ULM SOAM metric did not demonstrate a significant effect in the isocortex (Position: *F*_(2,45)_ = [0.359], *p* = 0.700; Genotype: *F*_(1,45)_ = [0.001], *p* = 0.981; Interaction: *F*_(2,45)_ = [0.109], *p* = 0.897), but was found to have a significant effect for genotype in the entorhinal cortex (Position: *F*_(2,45)_ = [1.179], *p* = 0.31; Genotype: *F*_(1,45)_ = [9.050], *p* = 0.004; Interaction: *F*_(2,45)_ = [0.066], *p* = 0.936) and hippocampus (Position: *F*_(2,45)_ = [1.307], *p* = 0.281; Genotype: *F*_(1,45)_ = [6.506], *p* = 0.014; Interaction: *F*_(2,45)_ = [0.229], *p* = 0.796).

For the 6-month cohort, there was a statistically significant effect between ULM vascularity and genotype for the isocortex (Position: *F*_(2,66)_ = [0.728], *p* = 0.487; Genotype: *F*_(1,66)_ = [8.248], *p* = 0.006; Interaction: *F*_(2,66)_ = [0.404], *p* = 0.669) and the entorhinal cortex (Position: *F*_(2,66)_ = 2.700, *p* = [0.075]; Genotype: *F*_(1,66)_ = 9.451, *p* = 0.003; Interaction: *F*_(2,66)_ = [0.591], *p* = 0.556). The hippocampus had statistical significance for both the animal genotype and imaging plane (Position: *F*_(2,66)_ = [10.05], *p* < 0.001; Genotype: *F*_(1,66)_ = [16.74], *p* < 0.001; Interaction: *F*_(2,66)_ = [2.477], *p* = 0.092). For ULM velocity there was a significant main effect for genotype, but not position, in the cortex (Position: *F*_(2,66)_ = [0.150], *p* = 0.861; Genotype: *F*_(1,66)_ = [42.27], *p* < 0.001; Interaction: *F*_(2,66)_ = [1.584], *p* = 0.213) and the entorhinal cortex (Position: *F*_(2,66)_ = [0.776], *p* = 0.465; Genotype: *F*_(1,66)_ = 20.92, *p* < 0.001; Interaction: *F*_(2,66)_ = [0.004], *p* = 0.996). For the hippocampus, ULM velocity was found to have a significant main effect for the bregma position and genotype (Position: *F*_(2,66)_ = [14.23], *p* < 0.001; Genotype: *F*_(1,66)_ = [14.69], *p* < 0.001; Interaction: *F*_(2,66)_ = [0.833], *p* = 0.439). The ULM SOAM index demonstrated a significant main effect for genotype in the isocortex (Position: *F*_(2,66)_ = [0.415], *p* = 0.662; Genotype: *F*_(1,66)_ = [30.66], *p* < 0.001; Interaction: *F*_(2,66)_ = [0.385], *p* = 0.682) and entorhinal cortex (Position: *F*_(2,66)_ = [0.954], *p* = 0.390; Genotype: *F*_(1,66)_ = [23.55], *p* < 0.001; Interaction: *F*_(2,66)_ = [0.336], *p* = 0.716). The SOAM was found to have significant main effect for imaging plane and genotype in the hippocampus (Position: *F*_(2,66)_ = [11.37], *p* < 0.001; Genotype: *F*_(1,66)_ = [6.500], *p* = 0.013; Interaction: *F*_(2,66)_ = [0.252], *p* = 0.777).

### Directional splitting of microbubble trajectories permits separate analysis of arteriole and venule flow in select regions

**Figure 4.**
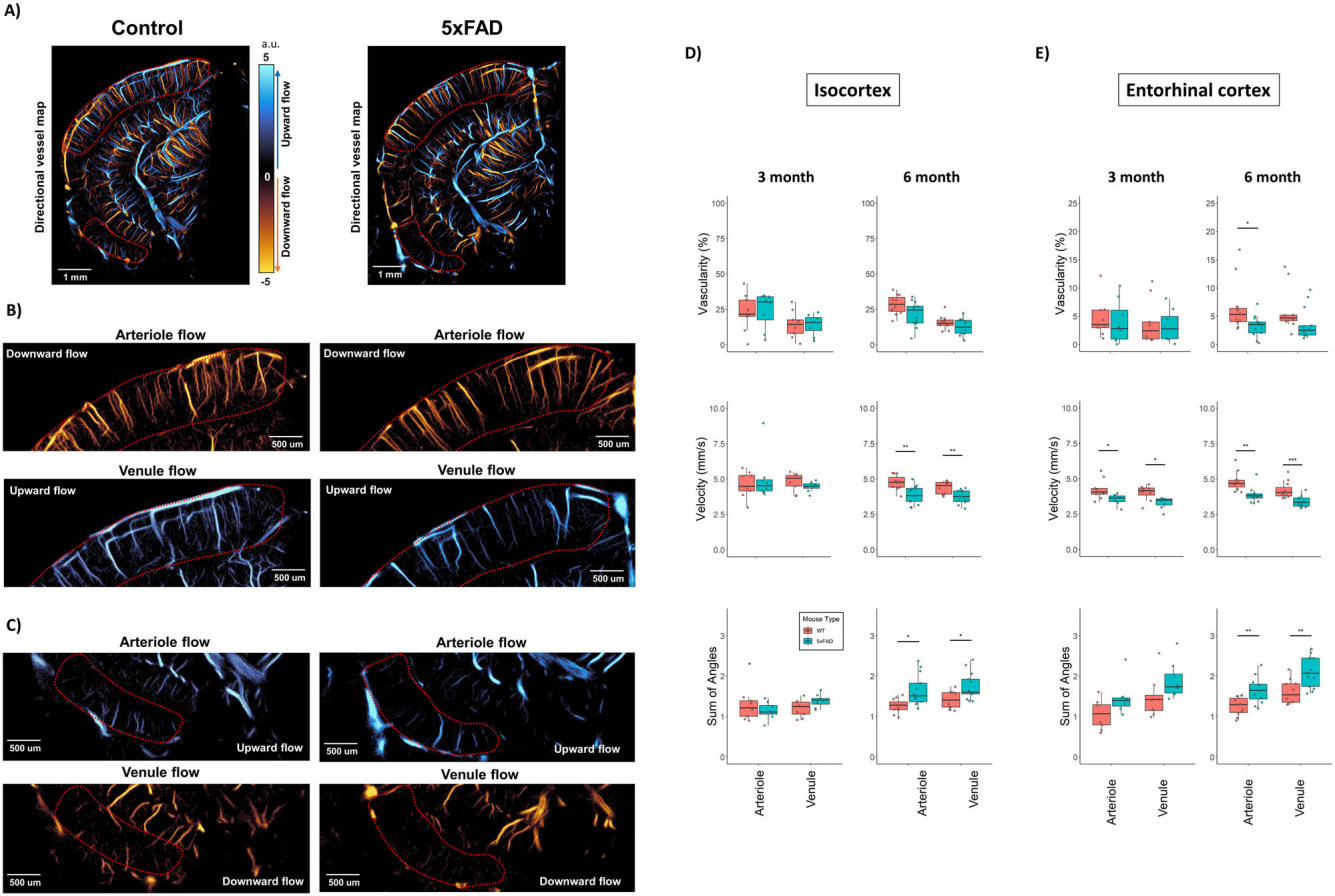
Separate arteriole and venule analysis in the cortical regions. ***A***, An example directional flow ULM map with ROI segmentation of the isocortex and entorhinal cortex. A magnified view of the isocortex (***B***) and entorhinal cortex (***C***) region demonstrate of separate arteriole and venule flow maps based on flow direction. Flow from superficial to deep into the cortex was considered as arteriole flow, and flow away was venule flow. ULM quantifications for both the arteriole and venule components are presented in (***D***) for the isocortex and (***E***) for the entorhinal cortex.

A feature of ULM image reconstruction is the ability to calculate the directional flow vector of microbubble trajectories, which can be displayed as a directional flow map (Fig. 4*A*). In select anatomical regions, such as the isocortex and entorhinal cortex, this directional information allows for the classification of flow and reconstructed vessels into arterial and venous components in an anatomical region of interest (Renaudin et al., 2022; Fig. 4*B,C*), under the assumption that movement from a large vessel to smaller branches is a characteristic of arterioles, and vice versa for venules. Thus, the independent analysis of the two directional maps gives insight into the relative contribution of arteriole versus venule flow to the AD pathology observed in the ULM quantifications. This is demonstrated in Figure 4*D,E* which analyzed the arteriole and venule contributions for the isocortex and entorhinal cortex, respectively.

For the isocortex (Fig. 4*D*), we found that there was no significant difference in the vascularization index in either the arteriole or venule components for the 3-month WT versus 5xFAD (Arteriole: WT = 22.9 ± 12.8% vs 5xFAD = 24.3 ± 12.6%, ANOVA *F*_(1,15)_ = [0.05], *p* = 0.827, *p*_corrected_ = 0.956; Venule: WT = 14.3 ± 9.3% vs 5xFAD = 14.1 ± 7.3%, ANOVA *F*_(1,15)_ = [0.003], *p* = 0.956, *p*_corrected_ = 0.956), the velocity (Arteriole: WT = 5.01 ± 0.73 mm/s vs 5xFAD = 5.36 ± 1.82 mm/s, ANOVA *F*_(1,15)_ = [0.289], *p* = 0.599, *p*_corrected_ = 0.599; Venule: WT = 5.15 ± 0.63 mm/s vs 5xFAD = 4.83 ± 0.36 mm/s, ANOVA *F*_(1,15)_ = [1.624], *p* = 0.222, *p*_corrected_ = 0.444), nor for SOAM (Arteriole: WT = 1.3 ± 0.43 a.u. vs 5xFAD = 1.14 ± 0.21 a.u., ANOVA *F*_(1,15)_ = [0.933], *p* = 0.349, *p*_corrected_ = 0.349; Venule: WT = 1.19 ± 0.21 a.u. vs 5xFAD = 1.39 ± 0.16 a.u., ANOVA *F*_(1,15)_ = [4.488], *p* = 0.051, *p*_corrected_ = 0.102). For the 6-month cohort, there was significance noted for the vascularization index of the arteriole flow for the uncorrected *p* value, but this was not found to be significant after correcting for multiple comparisons (WT = 28.5 ± 6.8% vs 5xFAD = 21.7 ± 8.7%, ANOVA *F*_(1,22)_ = [4.38], *p* = 0.048, *p*_corrected_ = 0.096), with no significance noted for the venule vascularization (WT = 15.6 ± 4.8% vs 5xFAD = 12.5 ± 5.7%, ANOVA *F*_(1,22)_ = [2.097], *p* = 0.162, *p*_corrected_ = 0.162). A significant difference was found for the velocity of both directions (Arteriole: WT = 5.12 ± 0.52 mm/s vs 5xFAD = 4.21 ± 0.64 mm/s, ANOVA *F*_(1,22)_ = [14.234], *p* < 0.001, *p*_corrected_ < 0.001; Venule: WT = 4.79 ± 0.41 mm/s vs 5xFAD = 4.05 ± 0.51 mm/s, ANOVA *F*_(1,22)_ = [14.84], *p* < 0.001, *p*_corrected_ < 0.001), as well as the SOAM (Arteriole: WT = 1.26 ± 0.17 a.u. vs 5xFAD = 1.65 ± 0.38 a.u., ANOVA *F*_(1,22)_ = [9.961], *p* = 0.005, *p*_corrected_ = 0.01; Venule: WT = 1.42 ± 0.21 a.u. vs 5xFAD = 1.72 ± 0.34 a.u., ANOVA *F*_(1,22)_ = [7.8], *p* = 0.011, *p*_corrected_ = 0.011).

For the entorhinal cortex (Fig. 4*E*), we found that there was no evidence of a vascularization index difference for the 3-month cohort (Arteriole: WT = 4.59 ± 3.31% vs 5xFAD = 3.94 ± 3.8%, ANOVA *F*_(1,15)_ = [0.142], *p* = 0.712, *p*_corrected_ = 0.752; Venule: WT = 3.85 ± 3.88% vs 5xFAD = 3.3 ± 2.8%, ANOVA *F*_(1,15)_ = [0.103], *p* = 0.752, *p*_corrected_ = 0.752), nor for the SOAM metric (Arteriole: WT = 1.06 ± 0.35 a.u. vs 5xFAD = 1.47 ± 0.44 a.u., ANOVA *F*_(1,15)_ = [4.445], *p* = 0.054, *p*_corrected_ = 0.108; Venule: WT = 1.48 ± 0.51 a.u. vs 5xFAD = 1.89 ± 0.45 a.u., ANOVA *F*_(1,15)_ = [2.846], *p* = 0.114, *p*_corrected_ = 0.114). There was a significant difference found for the velocity in both the arteriole and venule flows of the 3-month cohort (Arteriole: WT = 4.24 ± 0.7 mm/s vs 5xFAD = 3.53 ± 0.38 mm/s, ANOVA *F*_(1,22)_ = [5.768], *p* = 0.031, *p*_corrected_ = 0.031; Venule: WT = 3.99 ± 0.56 mm/s vs 5xFAD = 3.3 ± 0.4 mm/s, ANOVA *F*_(1,22)_ = [8.02], *p* = 0.013, *p*_corrected_ = 0.026). For the 6-month cohort, we found a significant difference in the vascularization index for the arteriole flow (WT = 6.55 ± 4.44% vs 5xFAD = 3.21 ± 1.86%, ANOVA *F*_(1,22)_ = [6.097], *p* = 0.022, *p*_corrected_ = 0.044), but not for the venous flow (WT = 5.91 ± 3.7% vs 5xFAD = 3.66 ± 2.78%, ANOVA *F*_(1,22)_ = [3.009], *p* = 0.097, *p*_corrected_ = 0.097). Significant differences were found for both flow directions for the velocity (Arteriole: WT = 4.8 ± 0.66 mm/s vs 5xFAD = 3.67 ± 0.53 mm/s, ANOVA *F*_(1,22)_ = [13.89], *p* < 0.001, *p*_corrected_ < 0.001; Venule: WT = 4.2 ± 0.59 mm/s vs 5xFAD = 3.38 ± 0.37 mm/s, ANOVA *F*_(1,22)_ = [17.29], *p* < 0.001, *p*_corrected_ < 0.001), and the SOAM metric (Arteriole: WT = 1.26 ± 0.22 a.u. vs 5xFAD = 1.65 ± 0.32 a.u., ANOVA *F*_(1,22)_ = [11.776], *p* = 0.003, *p*_corrected_ = 0.003; Venule: WT = 1.6 ± 0.29 a.u. vs 5xFAD = 2.08 ± 0.37 a.u., ANOVA *F*_(1,22)_ = [11.718], *p* = 0.002, *p*_corrected_ = 0.003).

Collectively, the vascular impairments seen in the 5xFAD animals, as measured by ULM, appear to be similar for the arteriole and venule components.

### ULM imaging enables deep brain individual vessel analysis

**Figure 5.**
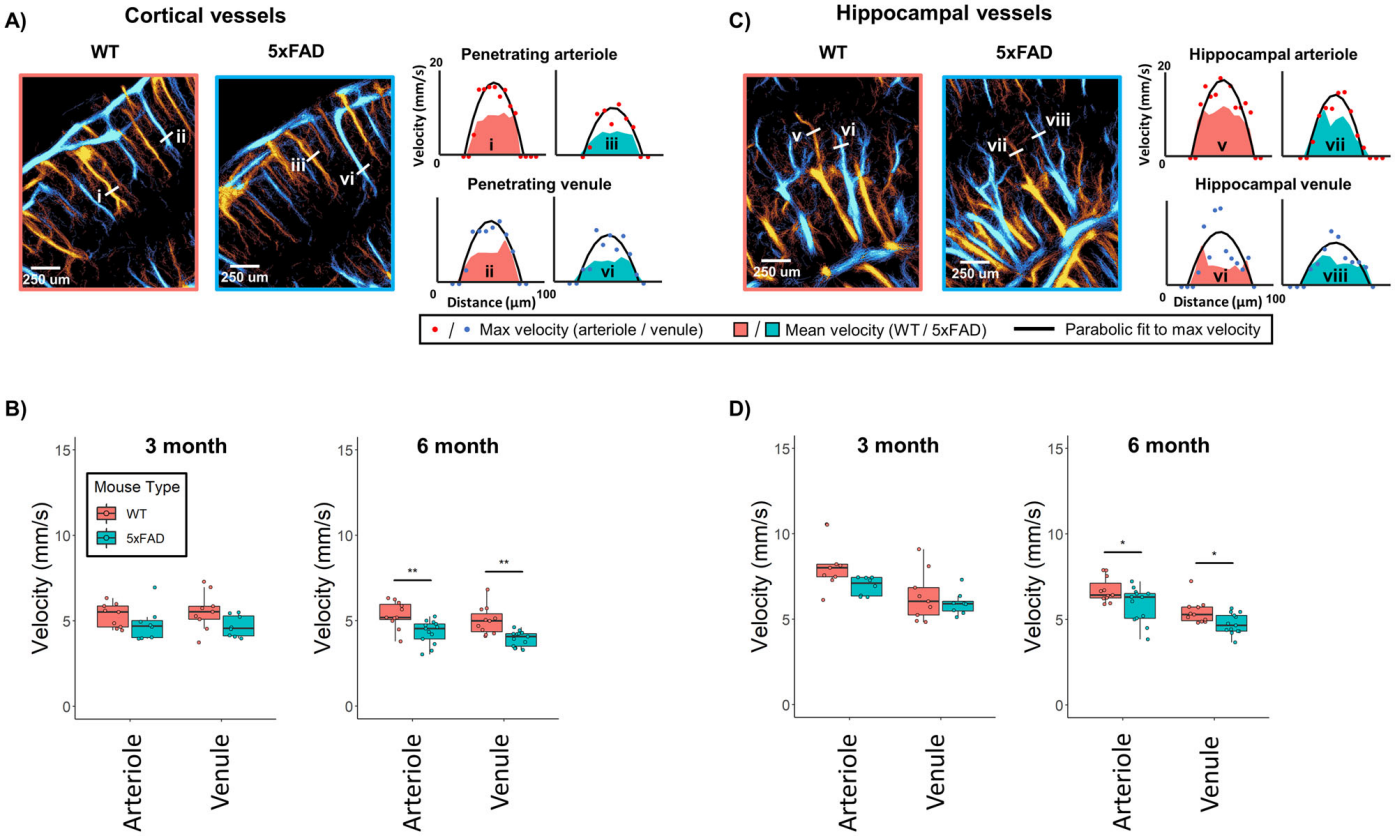
ULM individual vessel analysis. ***A***, Example ULM images from the cortex of representative WT/5xFAD animals with line profile segmentations of arterioles/venules, with corresponding velocity profiles demonstrated to the right. ***B***, Aggregate analysis of individual cortical vessels for both cohorts of animals. ***C***, Line profile segmentations of dorsal hippocampal vessels for the same animals, with corresponding velocity profiles. ***D***, The aggregate analysis of hippocampal arteriole and venule quantifications for both cohorts of animals.

Post-processing of the super-resolution vessel map allowed for velocity profile analysis of individual microvessels. As a case study for the investigational power of ULM, individual vessels from the cortex and the dorsal hippocampus were selected and analyzed from super-resolution reconstructions for WT and 5xFAD animals, as demonstrated in Figure 5. Velocity profiles were reconstructed for vessels of interest with both the lateral peak and mean velocity estimates, with a parabolic profile fit under the assumption of laminar blood flow. This process was repeated for all animals in this study, selecting arteriole/venule vessel cross-sections that were approximately 50 µm in diameter in all cases to control for the interaction(s) between vessel diameter and blood flow velocity. These results were then aggregated together, averaging velocity replicates taken from the same animal, and summarized in Figure 5.

The cortical vessel case study (Fig. 5*A*) shows that both the penetrating cortical arteriole (vessel i and iii) and penetrating cortical venule (vessel ii and iv) demonstrated a decrease in peak velocity for the 5xFAD animal relative to the WT control. This observation was also apparent in the aggregated results for cortical arteriole/venule quantifications (Fig. 5*B*) for the 6-month cohort (Arteriole: WT = 5.36 ± 0.77 mm/s vs 5xFAD = 4.33 ± 0.69 mm/s, ANOVA *F*_(1,22)_ = [11.87], *p* = 0.002, *p*_corrected_ = 0.002; Venule: WT = 5.01 ± 0.82 mm/s vs 5xFAD = 3.95 ± 0.44 mm/s, ANOVA *F*_(1,22)_ = [16.032], *p* < 0.001, *p*_corrected_ = 0.001). The 3-month cohort did not demonstrate a significant difference between WT and 5xFAD animals (Arteriole: WT = 5.31 ± 0.70 mm/s vs 5xFAD = 4.81 ± 0.98 mm/s, ANOVA *F*_(1,15)_ = [1.459], *p* = 0.246, *p*_corrected_ = 0.246; Venule: WT = 5.55 ± 1.10 mm/s vs 5xFAD = 4.68 ± 0.63 mm/s, ANOVA *F*_(1,15)_ = [3.888], *p* = 0.067, *p*_corrected_ = 0.134). These results are consistent with quantifications presented in Figure 4, which also demonstrated a decrease in both arteriole and venule flow for the 6-month 5xFAD cohort in comparison to WT.

The hippocampal vessel case study (Fig. 5*C*) also demonstrated a slight decrease in peak velocity for the 5xFAD animal relative to WT. The aggregate results (Fig. 5*D*) for the 3-month cohort demonstrated a trend toward a decrease in velocity for the 5xFAD animals (Arteriole: WT = 8.21 ± 1.46 mm/s vs 5xFAD = 6.94 ± 0.54 mm/s, ANOVA *F*_(1,15)_ = [5.392], *p* = 0.035, *p*_corrected_ = 0.070; Venule: WT = 6.35 ± 1.46 mm/s vs 5xFAD = 5.93 ± 0.68 mm/s, ANOVA *F*_(1,15)_ = [0.561], *p* = 0.465, *p*_corrected_ = 0.465). In the 6-month cohort, we found a slight significant decrease for both the arteriole and venule vessels in the 5xFAD animals in comparison to WT (Arteriole: WT = 6.71 ± 0.73 mm/s vs 5xFAD = 5.85 ± 1.02 mm/s, ANOVA *F*_(1,22)_ = [5.535], *p* = 0.028, *p*_corrected_ = 0.028; Venule: WT = 5.44 ± 0.70 mm/s vs 5xFAD = 4.74 ± 0.59 mm/s, ANOVA *F*_(1,22)_ = [6.900], *p* = 0.015, *p*_corrected_ = 0.028).

### ULM demonstrates functional changes prior to histological changes in vascular density

**Figure 6.**
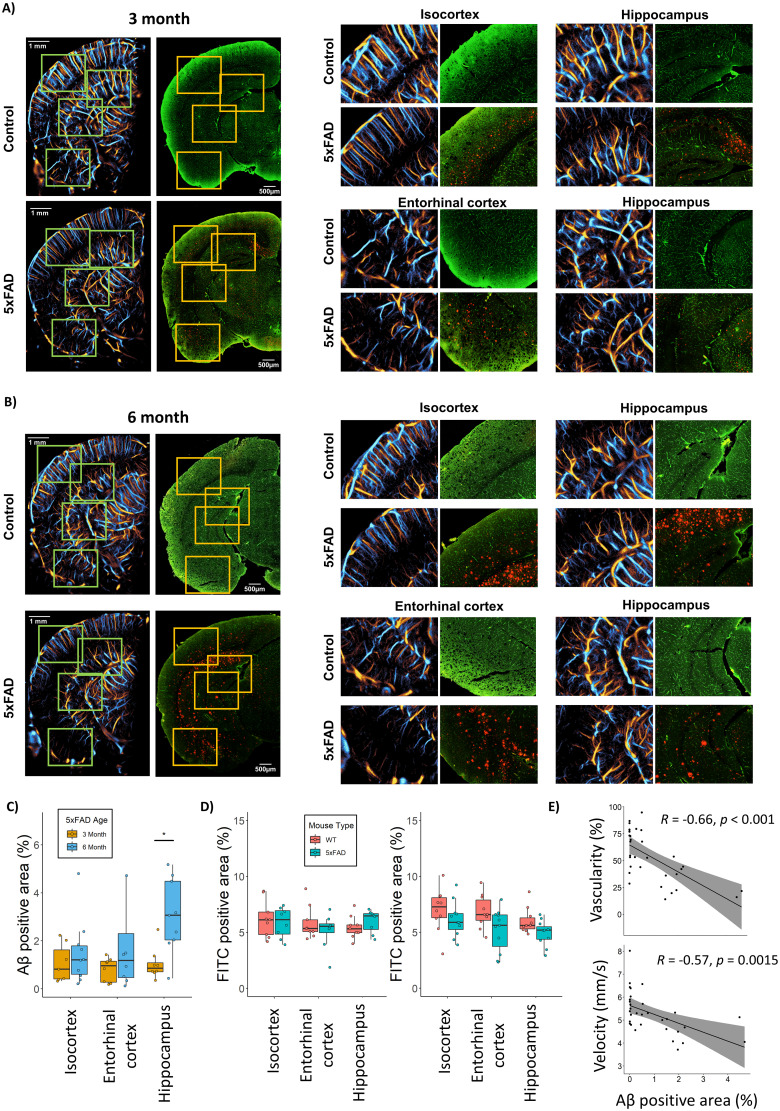
Comparison of ULM to histological analysis of vasculature and Aβ plaques. ***A***, This case study 3-month 5xFAD animal demonstrated a moderate degree of Aβ deposition (red channel) with evidence of mild ULM perfusion deficits in co-registered regions. ***B***, The 6-month 5xFAD exhibited heavy Aβ deposition and a profound vascular deficit on ULM imaging. ***C***, The 5xFAD cohort demonstrated a significant increase in Aβ deposition in the hippocampus, with an upward trend in all regions. ***D***, FITC vascular staining demonstrated no significant difference between the WT and 5xFAD animals in the 3-month cohort. A trend toward decreases in vascularity was seen in the isocortex and entorhinal cortex of the 6-month 5xFAD animals, with a no obvious trend in the hippocampus. ***E***, ULM quantifications from the case study animals demonstrated a significant negative correlation with Aβ deposition.

Co-registered ULM images with histological sections are demonstrated in Figure 6. It was found that there was a good overall correspondence between the FITC dextran staining on histology (green channel) and the ULM vascular reconstruction images. The WT animal had a high degree of vascularization throughout the entire brain observed with both ULM and histology, whereas the 5xFAD animal demonstrated a profound decrease in vasculature for the hippocampus and entorhinal cortex. Aβ plaque deposition is demonstrated in the red channel of the 5xFAD animal (Fig. 6*B*), with substantial levels of Aβ in the hippocampus and entorhinal cortex. The WT control did not show any evidence of Aβ deposition. Regions of high Aβ deposition showed a co-localization with reduced microvascular staining on histology and regionalized hypo-perfusion in the ULM images (Fig. 6*A,B*).

The summary analysis of histological sections for both Aβ deposition and FITC vascular staining are demonstrated in Figure 6*C,D*, respectively. The 5xFAD animals demonstrated a significant increase in Aβ deposition in the hippocampus between the 3-month cohort and 6-month cohorts (3-month = 1.03 ± 0.65% vs 6-month = 3.04 ± 1.54%, ANOVA *F*_(1,15)_ = [11.611], *p* = 0.004, *p*_corrected_ = 0.012), with a nonsignificant trend toward an increase in the entorhinal cortex (3-month = 0.79 ± 0.49% vs 6-month = 2.15 ± 2.66%, ANOVA *F*_(1,15)_ = [2.003], *p* = 0.179, *p*_corrected_ = 0.268), and no obvious trend for the isocortex (3-month = 1.06 ± 0.79% vs 6-month = 1.49 ± 1.35%, ANOVA *F*_(1,15)_ = [0.586], *p* = 0.456, *p*_corrected_ = 0.456). FITC vascular staining demonstrated no significant difference in vascular density for the WT and 5xFAD animals in the 3-month cohort in any region (IC: WT = 6.2 ± 1.6% vs 5xFAD = 5.7 ± 1.4%, ANOVA *F*_(1,15)_ = [0.372], *p* = 0.552, *p*_corrected_ = 0.552; EC: WT = 5.8 ± 1.46% vs 5xFAD = 5.1 ± 1.6%, ANOVA *F*_(1,15)_ = [0.893], *p* = 0.36, *p*_corrected_ = 0.54; HC: WT = 5.5 ± 1.0% vs 5xFAD = 5.9 ± 1.0%, ANOVA *F*_(1,15)_ = [1.159], *p* = 0.3, *p*_corrected_ = 0.54), which is consistent with the results demonstrated for ULM. The 6-month cohort demonstrated non-significant trends toward decreases in vascularity of the 5xFAD animals in comparison to the WT control in all regions, but these did not maintain significance following the correction for multiplicity. The FITC staining results for the isocortex were (WT = 7.4 ± 1.6% vs 5xFAD = 6.1 ± 1.7%, ANOVA *F*_(1,22)_ = [2.698], *p* = 0.121, *p*_corrected_ = 0.146), for the entorhinal cortex (WT = 7.7 ± 1.1% vs 5xFAD = 5.6 ± 1.9%, ANOVA *F*_(1,22)_ = [5.63], *p* = 0.035, *p*_corrected_ = 0.105), and the hippocampus (WT = 6.2 ± 1.3% vs 5xFAD = 5.3 ± 0.7%, ANOVA *F*_(1,22)_ = [2.397], *p* = 0.146, *p*_corrected_ = 0.146).

A correlation analysis was performed on the 5xFAD histological sections between Aβ positive area and FITC vascular staining for all brain regions. We found moderate-to-weak negative correlations for all regions: specifically, in the isocortex (*R* = −0.3, *p* = 0.095), the entorhinal cortex (*R* = −0.18, *p* = 0.34), and the hippocampus (*R* = −0.33, *p* = 0.049). A ULM-to-histology analysis was also performed on the sub-regions of the co-registered histological sections presented in Figure 6 to compare ULM metrics to Aβ deposition, which are summarized in Figure 6*E*. We found that both ULM vascularity and velocity were negatively correlated with Aβ positive staining (Vascularity: *R* = −0.66, *p* < 0.001; Velocity: *R* = −0.57, *p* = 0.0015).

## Discussion

This study investigated the vascular pathology in the 5xFAD mouse model of AD using super-resolution ULM imaging. We demonstrated that this technology provides microvascular-scale functional characterization of cerebrovascular impairments ranging from superficial to deep-brain structures, most notably for the hippocampus. This approach allows regional analysis of global shifts in vascular dynamics due to AD-related pathology while also permitting individual vessel characterization to elucidate localized impairments in function. ULM imaging also provides directional flow information that can decouple arterial and venous vascular features for select anatomical regions (e.g., dorsal penetrating cortical vessels), as demonstrated previously (Renaudin et al., 2022). Individual vessel velocity profile analysis was also demonstrated for hippocampal and cortical arterioles and venules, which yielded similar conclusions to the directional flow decoupling analysis.

We found that the 5xFAD mouse model demonstrated an early stage (3-month-old) decrease in hippocampal and entorhinal cortex median blood flow velocity, which progressed into deficits in vascular density in the 6-month-old animal cohort, along with sustained reductions in blood velocity for these regions (Fig. 2). We further demonstrated that this decrease in blood flow velocity was not exclusive to the hippocampus or entorhinal cortex: a local analysis of penetrating cortical vessels revealed a reduced peak velocity in 5xFAD mice relative to WT control (Fig. 5). Global quantifications from the isocortex also demonstrated a decrease in velocity in the 6-month cohort, but not in the younger cohort (Fig. 2). The analysis of arteriole and venule contributions to ULM quantifications (Fig. 4) found that the flow velocity in the entorhinal cortex was the most impacted vascular component in the 6-month cohort of 5xFAD animals, and that both the arterioles and venules showed functional deficits at the 3- and 6-month time points. This form of partitioned analysis may be critical for decoupling different aspects of AD pathology given the growing body of evidence that arterial and venular amyloid have distinct, and potentially synergistic, deposition mechanisms and impacts on cerebral amyloid angiopathy (Morrone et al., 2020). Histological quantification of vascular density (Fig. 6) confirmed the observation that a structural deficit in microvasculature was present in the older 5xFAD cohort but not the younger cohort. The SOAM, an established index of vascular tortuosity, was found to be elevated in the 6-month 5xFAD cohort in the isocortex and entorhinal cortex, leading to inefficient cerebral perfusion in 5xFAD mice. Together, these results demonstrate functional impairment of the vasculature at an early time point in the 5xFAD model, which only manifests as structural differences later in the development of the pathology, possibly due to aberrant or pathological vascular remodeling in response to regional stress. These findings can be contextualized with reports that AD-associated cognitive deficits in 5xFAD and other models of FAD are correlated to cerebral microvascular amyloid deposition but not to parenchymal amyloid plaques or to total Aβ levels (Xu et al., 2014). These findings, in conjunction with the ULM imaging results, suggest that early-stage cerebral microvascular changes lead to impaired cerebral perfusion and contribute to cognitive impairment.

These findings are consistent with a growing body of literature supporting the hypothesis that changes in microvascular integrity are a core finding in AD. For example, a recent analysis of biomarker data across platforms suggested that vascular biomarkers precede more traditional AD biomarkers during AD pathogenesis (Iturria-Medina et al., 2016). Further, some of the most common findings in the AD brain at autopsy are a decline in microvascular density, an increase in “string vessels” and an increase in blood vessel tortuosity (Bell and Ball, 1990; Challa et al., 2004; Richard et al., 2010; Hunter et al., 2012). Vascular decline may also contribute to more traditional pathological changes seen in the AD brain, such as the deposition of Aβ (Popa-Wagner et al., 1998; van Groen et al., 2005; Wang et al., 2010). Clinical evidence suggests that microvascular changes accelerate clinical decline in mild cognitive impairment and AD (Snowdon et al., 1997; Solfrizzi et al., 2004). Mechanistically, chronic microvascular compromise may lead to induction of cellular factors, such as hypoxia-inducible factor-1, leading to diminished cleavage of the amyloid precursor protein and therefore to Aβ deposition in or near blood vessels, which further limits blood supply, thus producing a vicious pathological cycle. The current data are supportive of the link between Aβ accumulation and microvascular compromise given the finding of a regional association between the two.

When compared to gold-standard histological analysis of co-registered brain sections, we found a good correspondence between vascular FITC staining and ULM maps of cerebral blood flow throughout the entire depth of the brain (Fig. 6), suggesting that ULM accurately reconstructs patent cerebrovasculature. Notably, Aβ plaque deposition was co-localized with regional hypo-perfusion in histological sections and in corresponding regions of ULM images (Fig. 6). This finding is consistent with similar findings in the literature which report that cerebral amyloid angiopathy, and the corresponding deficits in vascular perfusion and progression in microvascular damage, occur alongside the accumulation of Aβ in the 5xFAD model (Giannoni et al., 2016). Furthermore, a rosto-caudal analysis of ULM quantifications (Fig. 3) found a significant interaction effect for the hippocampal velocity and SOAM and imaging plane, which suggests that the largest difference between the cerebral vascular function of WT and 5xFAD animals is in the caudal portion of the hippocampus. This observation is supported by the finding that the caudal hippocampus has the highest burden of plaque (Forner et al., 2021). The different scale of the ULM vascularization index (Fig. 2) in comparison to the FITC positive area staining (Fig. 6), which differ by roughly an order of magnitude, should be noted. This can partially be explained by the vastly different sampling volumes between the ultrasound imaging and histological sectioning: the ultrasound beamwidth is several hundreds of micrometers whereas the histology was prepared as 40-μm-thick sections. Furthermore, ULM processing exclusively maps out the luminal volume of vasculature, where FITC-dextran staining generally indicates the vessel wall, and which can lead to an under-estimation bias relative to ULM.

This study has some additional limitations which should be discussed to better interpret the results presented. ULM reconstruction of microvasculature is inherently stochastic: vessels are perfused gradually, sparsely, and randomly by microbubbles through a process dictated by physiology and which necessitates long imaging durations to ensure adequate vascular sampling (Christensen-Jeffries et al., 2019; Hingot et al., 2019; Lowerison et al., 2020a). This has implications not only for the pragmatic challenges of the technology (e.g., difficulty with clinical translation, risk of animal death, obstacle to longitudinal study design), but also introduces several sources of variability into the microvascular quantifications extracted from ULM images. Tissue motion poses a persistent risk to super-resolution ULM reconstruction due to drift in microbubble localizations, which causes misregistration in the final accumulation. Although motion compensation was performed in the ULM processing pipeline, it is unable to correct for out-of-plane shifts or for tissue deformations (e.g., tissue motion from large vessel pulsatility). Furthermore, the long imaging duration may impart real physiological changes that can either obscure or exacerbate baseline differences in AD cerebrovasculature in comparison to WT animals. This study also used an open craniotomy for brain imaging, which can introduce significant changes to cerebrovascular hemodynamics. Previous ULM studies have been able to produce high quality reconstructions through intact skull (Nouhoum et al., 2021; Demeulenaere et al., 2022) of young mice (aged 7–14 weeks). Transcranial imaging of different age cohorts of 5xFAD animals with a high-frequency ultrasound probe should be investigated in future experiments, and would likely require substantial phase aberration correction for accurate microbubble localization through the thicker skulls of animals of the ages used in the current study. In addition, in this study, mice were anesthetized with vaporized isoflurane for a typical duration of about 2 h, a duration which impacts cardiac output of the animal and may also induce shifts cerebrovascular perfusion. In this context, we note that heart rate was not found to correlate with ULM metrics in this study, diminishing the possibility that differences in cardiovascular parameters explain FAD-related findings in this study. It has also been reported in literature that isoflurane has a dose-dependent dilatory effect on the cerebral blood flow (Matta et al., 1999), which may have differential consequences on 5xFAD mice and WT animals. Future work will need to examine whether similar FAD-related changes demonstrated in the current study are also seen in the absence of anesthesia. Finally, there is literature reporting sex-related differences in 5xFAD pathology (Forner et al., 2021; Poon et al., 2023). Although we found no significant difference between male and female animals for the 6-month cohort, it is possible that confounding sex-related differences in ULM measurements for the 3-month cohort, which had an imbalanced male:female ratio. Future studies should explicitly investigate this effect.

In conclusion, we have demonstrated the use of super-resolution ULM on the 5xFAD mouse model of AD (3- and 6-month-old cohorts), in comparison to age-matched WT controls, to reveal microvascular scale reconstructions throughout the whole brain depth. We found that functional decreases in hippocampal and entorhinal flow velocity preceded structural deficits in regional vascular density. We also found that the caudal portion of the hippocampus was the most impacted by vascular deficits found in the 6-month 5xFAD cohort. These data suggest that this novel imaging technology can be used to investigate functional AD pathophysiology *in vivo* and to evaluate novel therapeutic approaches that impact the deep microvasculature of mouse models of AD.
